# 2-Bromo-2-methyl-*N*-(4-methyl-2-oxo-2*H*-chromen-7-yl)propanamide

**DOI:** 10.1107/S1600536810026802

**Published:** 2010-07-14

**Authors:** N. Haridharan, V. Ramkumar, R. Dhamodharan

**Affiliations:** a Department of Chemistry, IIT Madras, Chennai, TamilNadu, India

## Abstract

In the title compound C_14_H_14_BrNO_3_, the coumarin ring system is almost planar (r.m.s. deviation = 0.008 Å) and an intra­molecular C—H⋯O inter­action generates an *S*(6) ring. In the crystal, mol­ecules are linked by N—H⋯O hydrogen bonds, with the C=O unit of the coumarin ring system acting as the acceptor group, generating [010] *C*(8) chains. The chain connectivity is reinforced by two C—H⋯O inter­actions.

## Related literature

For backgound to the properties of coumarin derivatives, see: Sinkel *et al.* (2008 ▶); Matyjaszewski *et al.* (2008 ▶); Stenzel-Rosenbaum *et al.* (2001 ▶); Thaisrivongs *et al.* (1994 ▶). For a related structure, see: Haridharan *et al.* (2010 ▶)
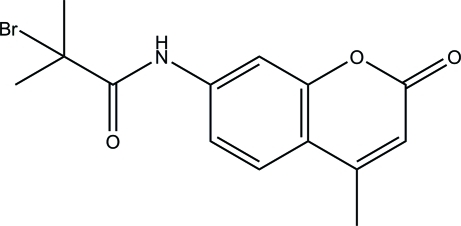


## Experimental

### 

#### Crystal data

C_14_H_14_BrNO_3_
*M* *_r_* = 324.17
Triclinic, 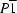

*a* = 6.7054 (8) Å
*b* = 9.2415 (11) Å
*c* = 11.7612 (15) Å
α = 105.255 (5)°
β = 100.630 (5)°
γ = 93.572 (5)°
*V* = 686.33 (15) Å^3^
*Z* = 2
Mo *K*α radiation
μ = 3.00 mm^−1^
*T* = 298 K
0.42 × 0.20 × 0.15 mm

#### Data collection

Bruker APEXII CCD diffractometer
Absorption correction: multi-scan (*SADABS*; Bruker, 2004 ▶) *T*_min_ = 0.366, *T*_max_ = 0.662
4624 measured reflections
2511 independent reflections
1716 reflections with *I* > 2σ(*I*)
*R*_int_ = 0.020

#### Refinement

*R*[*F* ^2^ > 2σ(*F* ^2^)] = 0.057
*wR*(*F* ^2^) = 0.174
*S* = 1.09
2511 reflections
179 parameters
1 restraint
H atoms treated by a mixture of independent and constrained refinement
Δρ_max_ = 1.16 e Å^−3^
Δρ_min_ = −0.53 e Å^−3^

### 

Data collection: *APEX2* (Bruker, 2004 ▶); cell refinement: *SAINT-Plus* (Bruker, 2004 ▶); data reduction: *SAINT-Plus*; program(s) used to solve structure: *SHELXS97* (Sheldrick, 2008 ▶); program(s) used to refine structure: *SHELXL97* (Sheldrick, 2008 ▶); molecular graphics: *ORTEP-3* (Farrugia, 1997 ▶); software used to prepare material for publication: *SHELXL97*.

## Supplementary Material

Crystal structure: contains datablocks global, I. DOI: 10.1107/S1600536810026802/hb5522sup1.cif

Structure factors: contains datablocks I. DOI: 10.1107/S1600536810026802/hb5522Isup2.hkl

Additional supplementary materials:  crystallographic information; 3D view; checkCIF report

## Figures and Tables

**Table 1:** Hydrogen-bond geometry (Å, °)

*D*—H⋯*A*	*D*—H	H⋯*A*	*D*⋯*A*	*D*—H⋯*A*
C8—H8⋯O3	0.93	2.21	2.804 (6)	121
N1—H1*N*⋯O2^i^	0.91 (2)	2.12 (2)	3.016 (5)	168 (5)
C6—H6⋯O2^i^	0.93	2.38	3.189 (6)	145
C13—H13*C*⋯O2^i^	0.96	2.51	3.347 (8)	146

Symmetry code: (i) 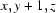.

**Fig. 1.**
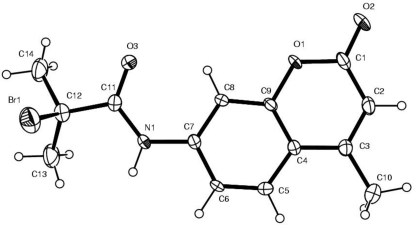
The molecular structure of (I) with atoms represented as 30% probability ellipsoids.

**Fig. 2.**
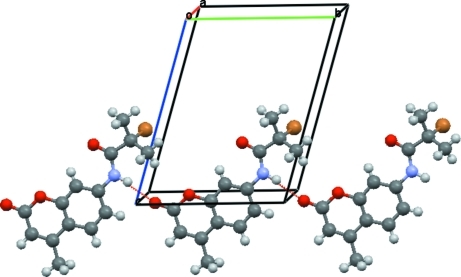
The packing diagram for (I) showing the N—H···O interaction along the b axis.

## supplementary crystallographic information

### Comment

The title compound C_14_ H_14_ Br N O_3_, is a monofunctional coumarin derivative, which is used as an initiator (Sinkel *et al.*, 2008) in Atom Transfer Radical Polymerization (ATRP). We have already reported a similar ATRP initiator (Haridharan *et al.*, 2010) with flourine containing coumarin derivative. The title compound reported here is a similiar derivative with bromo methyl propanamide and with a methyl substitution.

The synthesis of oxygen containing heterocyclic based initiators and their crystal structures are worth while to study due to their interesting properties and diverse bioactivities such as non peptidic HIV protease inhibition and tyrosine kinase inhibition (Thaisrivongs *et al.*, 1994).

In the title compound C_14_ H_14_ Br N O_3_, the coumarin ring system is plannar and the Br atom in the 2-bromo-2-methyl propanamide moiety is almost perpendicular to the ring.

The torsion angle of C6—C7—N1—C11 and C8—C7—N1—C11 are -177.89 (2)° and -2.75 (2)° respectively. The crystal is stabilized by intermolecular N—H···O hydrogen bond.

### Experimental

7-Amino-4-methylcoumarin (4 g, 0.022 moles), triethylamine (5.08 g, 0.050 moles) and THF (200 ml) were placed in a 3-neck round bottomed flask. Bromoisobutyrl bromide (11.54 g, 0.050 moles) was added slowly, using a syringe, with stirring, upon which an white precipitate of triethylammonium bromide was formed. The mixture was left to react for 6 h, with stirring. Subsequently, triethylammonium bromide, the precipitate was removed by filtration and the THF was removed by rotary evaporation. The resulting crude product was dissolved in ethyl acetate, washed with bicarbonate solution and then with water thrice followed by brine solution and dried over anhydrous sodium sulfate. The solvent was removed from the resulting solution by rotary evaporation. The product was purified by column chromatography technique using 10% ethyl acetate in hexane as the eluent to obtain pure initiator as a light yellow solid. Recrystallization of the compound from chloroform gave light yellow slabs of (I).

### Refinement

The nitrogen H atom was located in a difference Fourier map and refined isotropically. All other hydrogen atoms were fixed geometrically and allowed to ride on the parent carbon atoms, with aromatic C—H = 0.93 Å and methyl C—H = 0.96 Å. The displacement parameters were set for phenyl H atoms at *U*_iso_(H) = 1.2*U*_eq_(C) and methyl H atoms at *U*_iso_(H) = 1.5*U*_eq_(C).

### Crystal data

**Table:**

C_14_H_14_BrNO_3_	*Z* = 2
*M**_r_* = 324.17	*F*(000) = 328
Triclinic, *P*1	*D*_x_ = 1.569 Mg m^−^^3^
Hall symbol: -P 1	Mo *K*α radiation, λ = 0.71073 Å
*a* = 6.7054 (8) Å	Cell parameters from 1785 reflections
*b* = 9.2415 (11) Å	θ = 2.5–24.5°
*c* = 11.7612 (15) Å	µ = 3.00 mm^−^^1^
α = 105.255 (5)°	*T* = 298 K
β = 100.630 (5)°	Slab, light-yellow
γ = 93.572 (5)°	0.42 × 0.20 × 0.15 mm
*V* = 686.33 (15) Å^3^	

### Data collection

**Table:**

Bruker APEXII CCD diffractometer	2511 independent reflections
Radiation source: fine-focus sealed tube	1716 reflections with *I* > 2σ(*I*)
graphite	*R*_int_ = 0.020
phi and ω scans	θ_max_ = 28.3°, θ_min_ = 1.8°
Absorption correction: multi-scan (*SADABS*; Bruker, 2004)	*h* = −8→5
*T*_min_ = 0.366, *T*_max_ = 0.662	*k* = −11→10
4624 measured reflections	*l* = −11→14

### Refinement

**Table:**

Refinement on *F*^2^	Primary atom site location: structure-invariant direct methods
Least-squares matrix: full	Secondary atom site location: difference Fourier map
*R*[*F*^2^ > 2σ(*F*^2^)] = 0.057	Hydrogen site location: inferred from neighbouring sites
*wR*(*F*^2^) = 0.174	H atoms treated by a mixture of independent and constrained refinement
*S* = 1.09	*w* = 1/[σ^2^(*F*_o_^2^) + (0.1*P*)^2^ + 0.350*P*] where *P* = (*F*_o_^2^ + 2*F*_c_^2^)/3
2511 reflections	(Δ/σ)_max_ < 0.001
179 parameters	Δρ_max_ = 1.16 e Å^−^^3^
1 restraint	Δρ_min_ = −0.53 e Å^−^^3^

### Special details

**Table:**

Geometry. All e.s.d.'s (except the e.s.d. in the dihedral angle between two l.s. planes)are estimated using the full covariance matrix. The cell e.s.d.'s are takeninto account individually in the estimation of e.s.d.'s in distances, anglesand torsion angles; correlations between e.s.d.'s in cell parameters are onlyused when they are defined by crystal symmetry. An approximate (isotropic)treatment of cell e.s.d.'s is used for estimating e.s.d.'s involving l.s. planes.
Refinement. Refinement of *F*^2^ against ALL reflections. The weighted *R*-factor *wR* andgoodness of fit *S* are based on *F*^2^, conventional *R*-factors *R* are basedon *F*, with *F* set to zero for negative *F*^2^. The threshold expression of*F*^2^ > σ(*F*^2^) is used only for calculating *R*-factors(gt) *etc*. and isnot relevant to the choice of reflections for refinement. *R*-factors basedon *F*^2^ are statistically about twice as large as those based on *F*, and *R*-factors based on ALL data will be even larger.

### Fractional atomic coordinates and isotropic or equivalent isotropic displacement parameters (Å^2^)

**Table:**

	*x*	*y*	*z*	*U*_iso_*/*U*_eq_	
Br1	0.34908 (9)	0.90419 (7)	0.61854 (6)	0.0714 (3)	
C1	0.2652 (7)	0.2486 (5)	1.0477 (4)	0.0377 (11)	
C2	0.3283 (7)	0.3307 (5)	1.1731 (4)	0.0388 (11)	
H2	0.3551	0.2772	1.2299	0.047*	
C3	0.3497 (6)	0.4824 (5)	1.2107 (4)	0.0352 (10)	
C4	0.3086 (6)	0.5641 (5)	1.1221 (4)	0.0291 (10)	
C5	0.3260 (7)	0.7225 (5)	1.1480 (4)	0.0362 (11)	
H5	0.3639	0.7807	1.2279	0.043*	
C6	0.2890 (7)	0.7927 (5)	1.0596 (4)	0.0347 (10)	
H6	0.3042	0.8976	1.0795	0.042*	
C7	0.2285 (6)	0.7087 (5)	0.9391 (4)	0.0291 (9)	
C8	0.2092 (6)	0.5511 (4)	0.9098 (4)	0.0282 (9)	
H8	0.1700	0.4926	0.8301	0.034*	
C9	0.2497 (6)	0.4850 (4)	1.0021 (4)	0.0267 (9)	
C10	0.4152 (10)	0.5641 (7)	1.3424 (5)	0.0616 (15)	
H10A	0.3063	0.6170	1.3694	0.092*	
H10B	0.5336	0.6349	1.3549	0.092*	
H10C	0.4477	0.4925	1.3872	0.092*	
C11	0.1328 (7)	0.7309 (5)	0.7300 (4)	0.0394 (11)	
C12	0.0809 (8)	0.8434 (6)	0.6573 (4)	0.0464 (12)	
C13	−0.0004 (12)	0.9843 (7)	0.7192 (6)	0.0723 (18)	
H13A	−0.1170	0.9573	0.7491	0.108*	
H13B	−0.0397	1.0413	0.6629	0.108*	
H13C	0.1037	1.0443	0.7851	0.108*	
C14	−0.0590 (10)	0.7627 (8)	0.5376 (5)	0.0688 (17)	
H14A	−0.1897	0.7310	0.5509	0.103*	
H14B	−0.0003	0.6760	0.4984	0.103*	
H14C	−0.0757	0.8300	0.4876	0.103*	
N1	0.1878 (5)	0.7900 (4)	0.8526 (3)	0.0344 (9)	
O1	0.2284 (4)	0.3286 (3)	0.9663 (3)	0.0340 (7)	
O2	0.2428 (6)	0.1122 (4)	1.0081 (3)	0.0560 (10)	
O3	0.1211 (7)	0.5975 (4)	0.6804 (3)	0.0650 (12)	
H1N	0.196 (7)	0.891 (3)	0.889 (4)	0.048 (14)*	

### Atomic displacement parameters (Å^2^)

**Table:**

	*U*^11^	*U*^22^	*U*^33^	*U*^12^	*U*^13^	*U*^23^
Br1	0.0746 (4)	0.0678 (5)	0.0796 (6)	0.0004 (3)	0.0157 (3)	0.0365 (4)
C1	0.044 (2)	0.024 (3)	0.050 (3)	0.0048 (18)	0.007 (2)	0.020 (2)
C2	0.048 (2)	0.035 (3)	0.039 (3)	0.0044 (19)	0.0067 (19)	0.022 (2)
C3	0.041 (2)	0.034 (3)	0.032 (2)	0.0017 (18)	0.0041 (18)	0.014 (2)
C4	0.035 (2)	0.026 (2)	0.029 (2)	0.0043 (16)	0.0071 (16)	0.012 (2)
C5	0.049 (2)	0.026 (2)	0.028 (2)	0.0052 (18)	0.0016 (18)	0.002 (2)
C6	0.059 (3)	0.012 (2)	0.030 (3)	0.0029 (17)	0.0056 (19)	0.0035 (19)
C7	0.036 (2)	0.024 (2)	0.030 (2)	0.0050 (16)	0.0076 (16)	0.011 (2)
C8	0.038 (2)	0.018 (2)	0.026 (2)	0.0024 (16)	0.0035 (16)	0.0037 (19)
C9	0.0311 (18)	0.017 (2)	0.032 (2)	0.0040 (14)	0.0046 (16)	0.0070 (19)
C10	0.091 (4)	0.055 (4)	0.037 (3)	0.006 (3)	0.001 (3)	0.020 (3)
C11	0.055 (3)	0.035 (3)	0.031 (3)	0.009 (2)	0.0075 (19)	0.015 (2)
C12	0.060 (3)	0.041 (3)	0.042 (3)	0.008 (2)	0.008 (2)	0.018 (2)
C13	0.112 (5)	0.063 (4)	0.062 (4)	0.045 (4)	0.028 (3)	0.037 (3)
C14	0.080 (4)	0.074 (4)	0.051 (4)	−0.003 (3)	−0.012 (3)	0.036 (3)
N1	0.053 (2)	0.020 (2)	0.031 (2)	0.0074 (15)	0.0043 (16)	0.0105 (18)
O1	0.0520 (17)	0.0136 (15)	0.0361 (18)	0.0024 (12)	0.0046 (13)	0.0098 (14)
O2	0.088 (3)	0.0186 (18)	0.061 (2)	0.0042 (16)	0.0067 (19)	0.0166 (17)
O3	0.129 (4)	0.031 (2)	0.0283 (19)	0.016 (2)	0.0016 (19)	0.0069 (16)

### Geometric parameters (Å, °)

**Table:**

Br1—C12	2.017 (5)	C8—H8	0.9300
C1—O2	1.213 (5)	C9—O1	1.385 (5)
C1—O1	1.353 (6)	C10—H10A	0.9600
C1—C2	1.439 (7)	C10—H10B	0.9600
C2—C3	1.344 (6)	C10—H10C	0.9600
C2—H2	0.9300	C11—O3	1.208 (6)
C3—C4	1.438 (7)	C11—N1	1.370 (6)
C3—C10	1.502 (7)	C11—C12	1.529 (7)
C4—C9	1.377 (6)	C12—C13	1.503 (8)
C4—C5	1.407 (6)	C12—C14	1.514 (7)
C5—C6	1.358 (7)	C13—H13A	0.9600
C5—H5	0.9300	C13—H13B	0.9600
C6—C7	1.395 (6)	C13—H13C	0.9600
C6—H6	0.9300	C14—H14A	0.9600
C7—C8	1.397 (6)	C14—H14B	0.9600
C7—N1	1.414 (6)	C14—H14C	0.9600
C8—C9	1.373 (6)	N1—H1N	0.91 (2)
			
O2—C1—O1	116.6 (4)	H10A—C10—H10B	109.5
O2—C1—C2	125.3 (4)	C3—C10—H10C	109.5
O1—C1—C2	118.1 (4)	H10A—C10—H10C	109.5
C3—C2—C1	122.1 (4)	H10B—C10—H10C	109.5
C3—C2—H2	119.0	O3—C11—N1	123.0 (4)
C1—C2—H2	119.0	O3—C11—C12	120.7 (4)
C2—C3—C4	118.5 (4)	N1—C11—C12	116.2 (4)
C2—C3—C10	120.5 (4)	C13—C12—C14	111.2 (5)
C4—C3—C10	121.0 (4)	C13—C12—C11	116.9 (4)
C9—C4—C5	116.0 (4)	C14—C12—C11	109.7 (4)
C9—C4—C3	119.2 (4)	C13—C12—Br1	108.2 (4)
C5—C4—C3	124.8 (4)	C14—C12—Br1	106.0 (4)
C6—C5—C4	121.8 (4)	C11—C12—Br1	104.1 (3)
C6—C5—H5	119.1	C12—C13—H13A	109.5
C4—C5—H5	119.1	C12—C13—H13B	109.5
C5—C6—C7	120.5 (4)	H13A—C13—H13B	109.5
C5—C6—H6	119.7	C12—C13—H13C	109.5
C7—C6—H6	119.7	H13A—C13—H13C	109.5
C6—C7—C8	119.3 (4)	H13B—C13—H13C	109.5
C6—C7—N1	117.1 (4)	C12—C14—H14A	109.5
C8—C7—N1	123.5 (4)	C12—C14—H14B	109.5
C9—C8—C7	118.1 (4)	H14A—C14—H14B	109.5
C9—C8—H8	120.9	C12—C14—H14C	109.5
C7—C8—H8	120.9	H14A—C14—H14C	109.5
C8—C9—C4	124.2 (4)	H14B—C14—H14C	109.5
C8—C9—O1	114.9 (3)	C11—N1—C7	126.8 (4)
C4—C9—O1	120.9 (4)	C11—N1—H1N	122 (3)
C3—C10—H10A	109.5	C7—N1—H1N	111 (3)
C3—C10—H10B	109.5	C1—O1—C9	121.2 (3)
			
O2—C1—C2—C3	−179.9 (5)	C3—C4—C9—C8	−179.1 (4)
O1—C1—C2—C3	0.5 (6)	C5—C4—C9—O1	179.8 (3)
C1—C2—C3—C4	−0.2 (6)	C3—C4—C9—O1	0.5 (6)
C1—C2—C3—C10	179.8 (4)	O3—C11—C12—C13	150.9 (6)
C2—C3—C4—C9	−0.3 (6)	N1—C11—C12—C13	−27.8 (7)
C10—C3—C4—C9	179.7 (4)	O3—C11—C12—C14	23.1 (7)
C2—C3—C4—C5	−179.5 (4)	N1—C11—C12—C14	−155.6 (5)
C10—C3—C4—C5	0.5 (7)	O3—C11—C12—Br1	−89.9 (5)
C9—C4—C5—C6	−0.7 (6)	N1—C11—C12—Br1	91.4 (4)
C3—C4—C5—C6	178.6 (4)	O3—C11—N1—C7	−3.3 (7)
C4—C5—C6—C7	1.1 (7)	C12—C11—N1—C7	175.3 (4)
C5—C6—C7—C8	−1.0 (6)	C6—C7—N1—C11	177.9 (4)
C5—C6—C7—N1	178.4 (4)	C8—C7—N1—C11	−2.8 (6)
C6—C7—C8—C9	0.5 (6)	O2—C1—O1—C9	−180.0 (4)
N1—C7—C8—C9	−178.9 (4)	C2—C1—O1—C9	−0.3 (6)
C7—C8—C9—C4	−0.1 (6)	C8—C9—O1—C1	179.4 (4)
C7—C8—C9—O1	−179.7 (3)	C4—C9—O1—C1	−0.2 (5)
C5—C4—C9—C8	0.2 (6)		

### Hydrogen-bond geometry (Å, °)

**Table:**

*D*—H···*A*	*D*—H	H···*A*	*D*···*A*	*D*—H···*A*
C8—H8···O3	0.93	2.21	2.804 (6)	121
N1—H1N···O2^i^	0.91 (2)	2.12 (2)	3.016 (5)	168 (5)
C6—H6···O2^i^	0.93	2.38	3.189 (6)	145
C13—H13C···O2^i^	0.96	2.51	3.347 (8)	146

Symmetry codes: (i) *x*, *y*+1, *z*.
